# Modulating retinoid-X-receptor alpha (RXRA) expression sensitizes chronic myeloid leukemia cells to imatinib *in vitro* and reduces disease burden *in vivo*

**DOI:** 10.3389/fphar.2023.1187066

**Published:** 2023-05-31

**Authors:** Bharathi M. Rajamani, Raveen Stephen Stallon Illangeswaran, Esther Sathya Bama Benjamin, Balaji Balakrishnan, Daniel Zechariah Paul Jebanesan, Saswati Das, Aswin Anand Pai, Rakhi Thalayattu Vidhyadharan, Ajith Mohan, Sreeja Karathedath, Aby Abraham, Vikram Mathews, Shaji R. Velayudhan, Poonkuzhali Balasubramanian

**Affiliations:** ^1^ Department of Haematology, Christian Medical College, Vellore, India; ^2^ Department of Biotechnology, Thiruvalluvar University, Vellore, India; ^3^ Sree Chitra Tirunal Institute for Medical Sciences and Technology, Thiruvananthapuram, India; ^4^ Department of Integrative Biology, School of Bio Sciences and Technology, Vellore Institute of Technology, Vellore, India; ^5^ Manipal Academy of Higher Education, Manipal, India; ^6^ Centre for Stem Cell Research (CSCR), A Unit of InStem Bengaluru, Christian Medical College Campus, Vellore, India

**Keywords:** chronic myeloid leukemia (CML), imatinib, RXRA, RXRA ligands, resistance

## Abstract

**Introduction:** The ligand-activated transcription factors, nuclear hormone receptors (NHRs), remain unexplored in hematological malignancies except for retinoic acid receptor alpha (*RARA*).

**Methods:** Here we profiled the expression of various NHRs and their coregulators in Chronic myeloid leukemia (CML) cell lines and identified a significant differential expression pattern between inherently imatinib mesylate (IM)-sensitive and resistant cell lines.

**Results:** Retinoid-X-receptor alpha (*RXRA*) was downregulated in CML cell lines inherently resistant to IM and in primary CML CD34^+^ cells. Pre-treatment with clinically relevant RXRA ligands improved sensitivity to IM *in-vitro* in both CML cell lines and primary CML cells. This combination effectively reduced the viability and colony-forming capacity of CML CD34^+^ cells *in-vitro. In-vivo,* this combination reduced leukemic burden and prolonged survival. Overexpression (OE) of *RXRA* inhibited proliferation and improved sensitivity to IM *in-vitro*. *In-vivo, RXRA* OE cells showed reduced engraftment of cells in the bone marrow, improved sensitivity to IM, and prolonged survival. Both *RXRA OE* and ligand treatment markedly reduced BCR::ABL1 downstream kinase activation, activating apoptotic cascades and improving sensitivity to IM. Importantly, RXRA OE also led to the disruption of the oxidative capacity of these cells.

**Conclusion:** Combining IM with clinically available RXRA ligands could form an alternative treatment strategy in CML patients with suboptimal response to IM.

## 1 Introduction

Chronic myeloid leukemia (CML) is a myeloproliferative neoplasm characterized by a reciprocal chromosomal translocation t(9;22) that generates the BCR::ABL1 fusion protein, a constitutively active tyrosine kinase essential for the pathogenesis of the disease. Molecular targeted therapy with tyrosine kinase inhibitors (TKI) such as imatinib mesylate (IM) has dramatically improved the outcome of this disease. However, a proportion of patients fail to achieve or retain molecular response with TKIs leading to disease progression.

The mechanism of TKI resistance in CML has been extensively studied and broadly classified as BCR::ABL1 dependent and independent. BCR::ABL1-dependent mechanisms include mutations in the BCR::ABL1 kinase domain and over expression of BCR::ABL1 or gene expression. BCR::ABL1 independent resistance mechansim include the persistance of leukemic stem cells, activation of multiple signaling pathways, and epigenetic events (Milojkovic and Apperley, 2009; Dohse et al., 2010; Patel et al., 2017; Loscocco et al., 2019). Mutations in the BCR::ABL kinase domain are seen in ∼30% of CML patients with suboptimal responses to imatinib. Some patients fail multiple TKIs without having documented kinase domain mutations. Effectively targeting these TKI-insensitive cells by combination therapies that interfere with various signaling pathways has been attempted previously (Prost et al., 2015; Irvine et al., 2016; Wagle et al., 2016; Agarwal et al., 2017; Finch et al., 2017). Although combining repurposed clinical agents such as venetoclax (Ko et al., 2014; Carter et al., 2016), pioglitazone (Prost et al., 2015), and tigecycline (Kuntz et al., 2017) with TKIs are shown to eliminate leukemic stem cells (LSCs) selectively, these drugs are not translated into clinics for CML treatment. Targeting nuclear hormone receptors (NHRs) with specific ligands has improved disease outcomes and survival in solid tumors (Culig, 2014; Wong et al., 2014; de Bono et al., 2018; Rathkopf et al., 2018; Smith et al., 2018; Munster et al., 2019). However, except for the role of all-trans retinoic acid (ATRA) in acute promyelocytic leukemia (APL) (Grignani et al., 1998; Lengfelder et al., 2005; Zheng et al., 2005; Park et al., 2008), no studies show the role of NHRs in modulating imatinib resistance in CML cells.

NHRs are a family of ligand-dependent transcription factors frequently dysregulated in various cancer types, rendering this family of proteins potential therapeutic targets for cancers (Petrie et al., 2007; Feldman et al., 2014; Quintero Barceinas et al., 2015). NHR ligands bind to the ligand binding domain of the receptor, which dissociates the co-repressors from the receptor and facilitates the binding of the co-activator for receptor activation (Sonoda et al., 2008). NHR ligand treatments improved disease outcomes with limited toxicity in various solid tumors. The most widely explored NHRs are estrogen (ER) and androgen receptor (AR), and their ligands are used as monotherapy or combination therapy for breast and prostate cancer. Long-term treatment of ER ligand tamoxifen in patients with breast cancer reduced disease mortality and death compared to no tamoxifen therapy (Group EBCTCG EBCTC, 2011; Davies et al., 2013). AR ligands (Flutamide, Bicalutamide, Enzalutamide, and Apalutamide) increased metastasis-free survival in patients with metastatic prostate cancer (Culig, 2014; Wong et al., 2014; de Bono et al., 2018; Rathkopf et al., 2018; Smith et al., 2018). Glucocorticoid receptor (GR) ligand, Relacorilant in combination with Nab-paclitaxel improved outcomes with limited toxicity in patients with adenocarcinoma and ovarian cancer (Munster et al., 2019). Retinoid-X-receptor alpha (*RXRA*) ligand bexarotene in combination with chemotherapy was more effective associated with better survival, and improved response in refractory cutaneous T-cell lymphoma (CTCL) (Duvic et al., 2001a; Duvic et al., 2001b; Pileri et al., 2013), metastatic breast cancer (Esteva et al., 2003; Yen et al., 2004; Yen and Lamph, 2005) and non-small cell lung carcinoma (NSCLC) (Khuri et al., 2001; Edelman et al., 2005). The role of NHRs in myeloid malignancies is relatively less explored except for the use of RARA (retinoic acid receptor alpha) ligand all-trans retinoic acid (ATRA) as a differentiation agent in APL. Preliminary results from our laboratory suggest that NHRs such as *PPARG* and *RXRA* were downregulated in chemoresistant acute myeloid leukemia (AML) cell lines (Karathedath et al., 2013), suggesting that NHRs can be modulated to improve chemosensitivity.

There are limited reports on the use of RXRA ligands in hematological malignancies. Rexinoid-X-receptors (RXR) are members of the NHR superfamily (Evans and Mangelsdorf, 2014). 9-cis retinoic acid, 9 cis-13,14-Dihydroretinoic acid, and long-chain fatty acids are natural ligands for RXR (Arnold et al., 2012; Jones et al., 2015; Rühl et al., 2015; Niu et al., 2017). In mammalian cells, three genes code for three RXR isoforms- *RXRA*, *RXRB*, and *RXRG* (Nohara et al., 2009). *RXRs* either homodimerize or heterodimerize with other NHRs for transcriptional activation. The heterodimeric partners of *RXRs* include retinoic-acid receptor (*RAR*), vitamin-D-receptor (*VDR*), peroxisome proliferator-activated receptors (*PPAR*), liver-X-receptor (*LXR*), pregnane-X-receptor (*PXR*), and the constitutive androsterone receptor (*CAR*) (Bugge et al., 1992; Kliewer et al., 1992; Willson and Kliewer, 2002; Calkin and Tontonoz, 2012). *RARA* and *RXRA* expression is downregulated in AML cells compared to myelodysplastic syndrome (Welch et al., 2014). Several studies have shown the efficacy of bexarotene as a pan-RXR ligand in AML. Bexarotene treatment induced differentiation and apoptosis in AML blast cells *in-vitro* and in AML cell lines (Altucci et al., 2005; Sanchez et al., 2014). AML patients treated with bexarotene showed differentiation *in-vivo* (Tsai et al., 2008). Bexarotene, combined with decitabine, was well tolerated and showed a modest response in older relapsed AML patients (Welch et al., 2014). The dual activation of RXRA-RARA heterodimers by the specific ligands, ATRA and bexarotene effectively targeted MLL-AF9 myelomonocytic leukemic cells *in-vitro* and improved the survival *in-vivo* in mice models (Di Martino et al., 2021a). DT448/9PP mutated *RXRA* receptor overexpression enhanced transcriptional activity leading to differentiation and loss of colony-forming ability in KMT2A-MLLT3 transformed AML cells (Di Martino et al., 2021b). Bexarotene treatment increased the protein expression of RXR isoforms (*RXRA*, *RXRB* & *RXRG*) in SH-SY5Y cells (Dheer et al., 2018). Bexarotene treatment increased the protein expression of *RXR* and *PPARG* in brain cells (Zuo et al., 2019). RXRA ligand treatment increased the mRNA expression of other NHRs, such as *PPARD*, *LXRA*, & *LXRB* (Di Martino et al., 2021a), in MLL-AF9 AML mice *in-vivo*.

Few studies have addressed the role of NHRs in modulating TKI sensitivity in CML. PPARG agonist pioglitazone was reported to target CML cells effectively by down-regulating transcription factors such as STAT5, CITED2, and HIF2a, which are overexpressed in CML stem cells (Prost et al., 2015). PPARG ligand-thiazolidinedione has been reported to synergize with TKIs and target CML stem cells (Prost et al., 2015; Glodkowska-Mrowka et al., 2016). CML patients treated with pioglitazone combined with imatinib achieved a deeper molecular response of MR4.5 at 12 months (Rousselot et al., 2017). However, the role of other NHRs and the effect of their ligands in overcoming inherent TKI resistance in CML is still not completely understood.

We investigated the differential expression profile of NHRs in CML cell lines that are inherently imatinib sensitive vs. resistant. We also assessed the effect of RXRA ligand treatment combined with imatinib on improving TKI sensitivity in CML cell lines, primary cells *in-vitro,* and in a CML cell line-derived xenograft mice model.

## 2 Materials and methods

### 2.1 Chemicals and reagents

Imatinib Mesylate (SML1027), methyl thiazolyldiphenyl-tetrazolium bromide (MTT), 9-cis retinoic acid (9cRA), Bexarotene (Bexa), acitretin (ACI), SR11237, all trans retinoic acid (ATRA), testosterone, 17β-estradiol (17βE), pioglitazone (PIO), rosiglitazone (rosi), Triiodothyronine (T3) and GSK4716 were from Sigma-Aldrich (St Louis, MO, United States). Rabbit monoclonal p-CRKL (3,181), p-STAT5 (9,351), p-AKT (4,060), caspase-3 (9,661), PARP (5,625), and RXRA (3,085) were from Cell Signaling Technologies (Danvers, MA, United States). β-actin (A5441) was from Sigma-Aldrich (St. Louis, MO), and horseradish peroxidase-conjugated goat anti-rabbit IgG (1:10,000), goat anti-mouse IgG (1:10,000) from Cell Signaling Technologies (Danvers, MA, United States). Imatinib Mesylate used for mice model experiments is a gift from NATCO, India.

### 2.2 Cell lines and primary cells

CML cell lines JURL-MK1, KCL22, Lama84, KYO1, EM2, and KU812, were a kind gift from Dr. Markus Muschen, University of California San Diego (UCSD), with approval from DSMZ, Germany. Cell lines were cultured in RPMI medium with 10%-FBS and antibiotics (100 U/ml-penicillin and 100 μg/mL-streptomycin) in a humidified atmosphere with 5% CO_2_ at 37°C.

Bone marrow or peripheral blood samples were collected after obtaining written informed consent from imatinib naïve adult CP-CML patients at diagnosis before the initiation of therapy. This study was approved by the Institutional Review Board of Christian Medical College, Vellore (IRB Min No. 8704). Primary CML cells from blood or bone marrow were processed and enriched for CML CD34^+^ cells, as reported previously (Rajamani et al., 2020).

### 2.3 RNA extraction and cDNA synthesis

Total RNA was extracted from CML cell lines (JURL-MK1, KU812, KYO1, EM2, Lama84, and KCL22) and treated with or without NHR ligands using Trizol reagent. RNA (1 μg) was converted to cDNA using random hexamer and reverse transcriptase enzyme (High-capacity cDNA synthesis kit, Thermo Scientific).

### 2.4 NHR profiling

To identify the basal expression of NHRs and coregulators in CML cell lines, we used an NHR RT^2^ PCR array (SA Biosciences, Germany) (https://geneglobe.qiagen.com/product-groups/rt2-profiler-pcr-arrays). The expression of each NHR was normalized using five housekeeping genes (*ACTB*, *GAPDH*, *B2M*, *HGPRT* & *RPLP0*), as recommended by the manufacturers. The data was analyzed using SA biosciences web-based analysis software (www.sabiosciences.com/pcr/arrayanalysis.php).

### 2.5 q-RT-PCR analysis of NHR expression

The expression of *AHR* (TaqMan assay ID: Hs00169233_m1), *AR* (Hs00171172_m1), *ESR1* (Hs01046816_m1), *ESRRG* (Hs00976243_m1), *PPARG* (Hs01115513_m1), *RXRA* (Hs0107640_m1), *RXRB* (Hs 00232774_m1) and *THRA* (Hs00268470_m1) were evaluated by qRT-PCR. Imatinib influx transporter *SLC22A1*/*hOCT1* (HS00427554-m1) expression was measured using TaqMan assay after treating the primary CML cells with NHR ligands. *GAPDH* was used as the housekeeping gene for these targets. Fold change in the expression of other NHRs such as *RARA*, *PPARG,* and *VDR* was measured by RQ-PCR using SYBR green chemistry (SYBR premix Ex TaqII (Tli RNAseH Plus), Takara, Bio, Shiga, Japan), and *ACTB* was used as the housekeeping gene to calculate the relative expression (∆∆Ct) of these targets.

### 2.6 In-vitro-cytotoxicity assay

CML cell lines JURL-MK1, KCL22, Lama84, KYO1, EM2, and KU812 [1 × 10^5^], were treated in a 96-well plate with increasing concentrations of imatinib (0–500 nM) for 48 h. Cell viability was measured at standard wavelength 570 nm and reference wavelength 630 nm using EL800 microplate reader with KC junior software (Biotek Winooski, VT, United States).

CML cell lines (KCL22 & Lama84) were pretreated with NHR agonists (agonists and their concentrations as listed in Supplementary Table S1) for 24 h, followed by treatment with increasing concentration of imatinib (0 nM–500 nM). All the experiments were performed in triplicates. The inhibitory concentration of 50% (IC-50) was calculated using Adapt software.

As suggested by previous reports (Austin et al., 2015; Wang et al., 2017), bulk primary CML cells [3 × 10^5^] were pretreated with NHR agonists for 24 h, followed by treatment with increasing concentration (0–100 uM) of imatinib for 48 hrs.

### 2.7 RNA expression by nanostring

RNA was extracted from CML CD34^+^ cells (*n* = 39) and healthy donor CD34^+^ cells (*n* = 9) using total RNA purification plus kit (Norgen Biotek Corp, Canada). Expression of *RXRA* was assessed using nCounter, and data counts were normalized with housekeeping genes–*ACTB*, *GAPDH* & *GUS* using nSolver software (NanoString, Technologies, Seattle, WA).

### 2.8 Colony forming unit assay

Primary CML (bulk & CD34^+^) or healthy donors (peripheral blood mononuclear cells—PBMNCs & CD34^+^) cells (2 × 10^5^) were treated with and without RXRA ligands 9-cis retinoic acid, acitretin, or bexarotene in combination with imatinib for 24 h; 1 × 10^4^ cells were seeded in methylcellulose medium (MethoCult™ H3334 classic, StemCell Technologies, Vancouver, Canada). Colonies were visualized using a phase-contrast microscope (×10 objective) and enumerated after 14 days.

### 2.9 Apoptosis assay

Primary CML CD34^+^ cells (2 × 10^5^) were treated with RXR ligands 9-cis retinoic acid, acitretin, or bexarotene for 24 h, followed by imatinib for 48 h. Cells were washed with 1XPBS and stained with Annexin-V conjugated with allophycocyanin (APC) and 7-Aminoactinomycin D (7AAD) (BD-bioscience, San Diego, CA). BD-Accuri C6 (BD Biosciences, Franklin Lakes, NJ) with BD Accuri C6 software (Version 1.0.264.21) was used for flow cytometry analysis.

### 2.10 Western blot analysis for BCR-ABL downstream signaling pathway

CML cell lines (KCL22, Lama84) or primary CML cells were treated with RXRA ligands (9-cis retinoic acid, acitretin, or bexarotene) for 24 h, followed by imatinib for 48 h. The whole-cell lysate was prepared using radioimmunoprecipitation assay (RIPA) buffer supplemented with protease-phosphatase inhibitor cocktail (Roche, IN, United States) and 2 mM phenyl methyl sulfonyl fluoride (Sigma Aldrich). Total protein concentration was measured using the Bradford assay. 50 ug of protein was resolved in a 10% SDS PAGE. Anti-p-CRKL, anti-p-AKT, anti-p-STAT5, anti-caspase3, and anti-PARP antibodies were used, and β-actin was used as the loading control.

### 2.11 Overexpression of RXRA in CML cell line

The RXRA plasmid containing the full-length cDNA (TFORF2199) was purchased from Addgene (Addgene plasmid #141967; RRID: Addgene_141967). Lentivirus particles were prepared by transfecting 293T cells with the RXRA cDNA plasmid and packaging plasmids. Lentiviruses were collected after 48, 60, and 72 h, pooled together, filtered and concentrated using a lenti-X concentrator (Takara, Bio, Shiga, Japan), and stored at –80 °C until use. Lama84 cells were transduced with lentiviruses in the presence of polybrene, followed by spinfection. RXRA OE virus transduced Lama84 cells were selected by puromycin (1 μg/mL).

### 2.12 CML cell line-derived xenograft (CDX) mice model

293T cells were transfected with plasmid lentiX-Luc2 containing a luciferase reporter for lentivirus production. KCL22 cells were transduced with lentivirus containing the luciferase reporter. Luciferase transduced KCL22 (1*10^6^ cells) were then transplanted into sub-lethally irradiated (2.5Gy) 8–10 weeks old NSG mice (Jackson Laboratory, United States) via tail-vein. Disease development was monitored by measuring luciferase expression by injecting D-luciferin substrate and imaging using an IVIS Spectrum *in-vivo* imaging system (PerkinElmer, United States). One week after transplantation, the mice were treated orally with either imatinib mesylate (100 mg/kg dissolved in water) (NATCO-Pharma Limited, India) or acitretin (10 mg/kg dissolved in corn oil) or in combination for 21 days. Engraftment of KCL22 cells in the mice was assessed by measuring luciferase expression every week for 6–7 weeks. All animal experiments were carried out per protocols approved by the Institutional Animal Care and Use Committee (IAEC No. 5/2019).

RXRA/empty vector transduced Lama84 (2*10^6^) cells were transplanted into sub-lethally irradiated (2.5Gy) 8–10 weeks old NSG mice via tail-vein. The leukemic burden in peripheral blood was assessed using flow cytometry by measuring human CD45 expression (BD-bioscience, CA). The percentage of survival of mice was evaluated by Kaplan-Meir survival analysis.

### 2.13 Extracellular flux analysis using seahorse XFe24 analyzer

Oxygen consumption rate (OCR) and extracellular acidification rate (ECAR) were measured using a Seahorse-XFe24 Analyzer (Agilent Technologies). Briefly, Lama84 EV and RXRA OE 4 × 10^5^cells per well (4 replicates) were seeded in a 24-well XF24 plate coated with retronectin (Takara Bio, Shiga, Japan). Thirty minutes before analysis, the medium was replaced with Seahorse XF media (Agilent Technologies, Santa Clara, CA, United States), and the plate was incubated at 37°C. Three sequential measurements of OCR and ECAR were taken at basal, proton leak (oligomycin), maximal respiration (FCCP), and OXPHOS inhibition (rotenone and Antimycin-A) to assess bioenergetics.

### 2.14 Statistical analysis

Paired *t*-test, non-parametric *t*-test, and ANOVA with Kruskal-Wallis’s correction were used where appropriate. All statistical analysis were done using GraphPad Prism-v5 software, and a *p*-value *<* 0.05 was considered statistically significant.

## 3 Results

### 3.1 Differential expression profile of NHRs in imatinib sensitive vs. resistant CML cell lines

**FIGURE 1:**
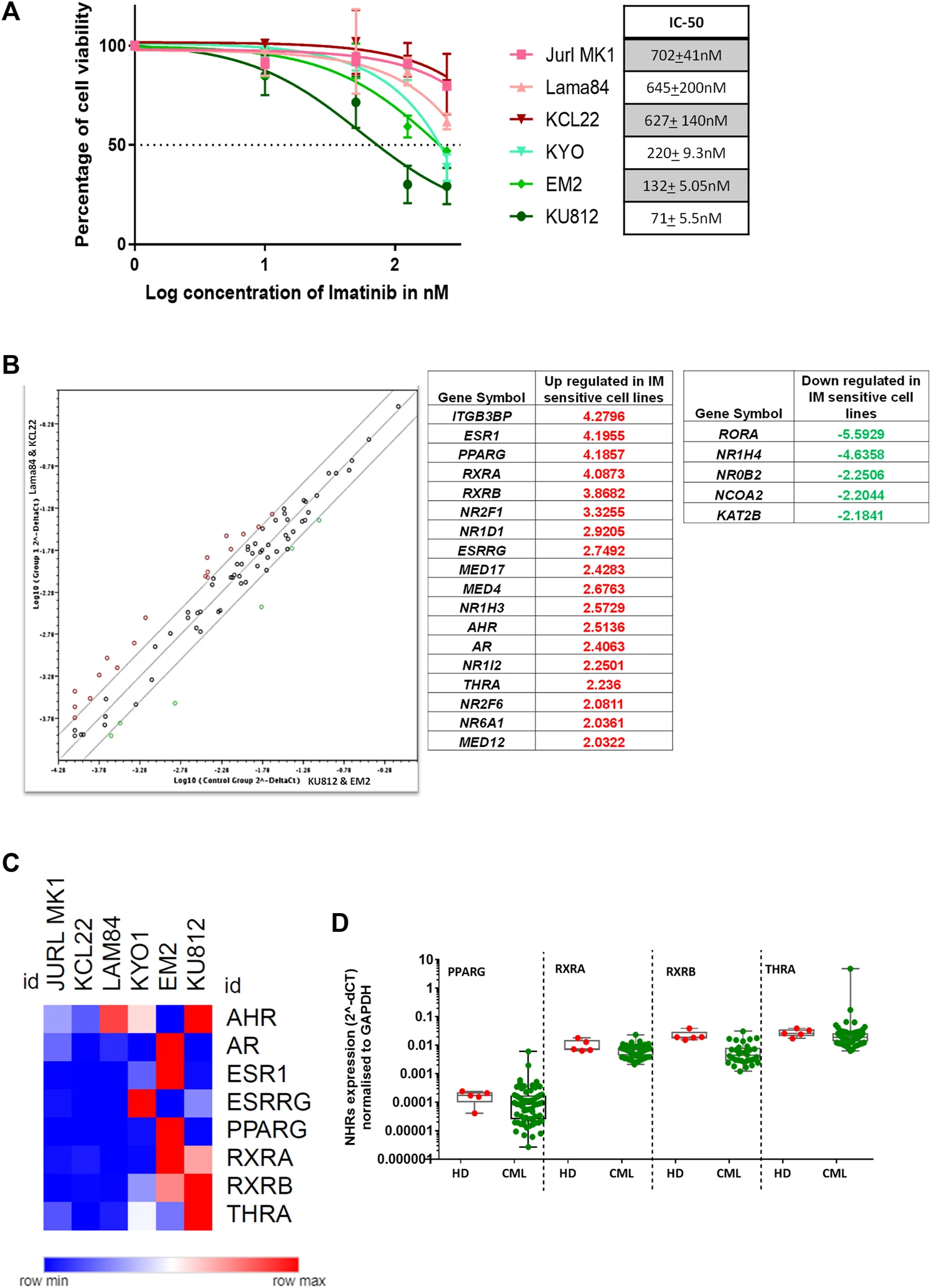
Nuclear hormone receptors and coregulators show differential expression in imatinib sensitive vs. resistant CML cell lines **(A)** Percentage cell viability in CML cell lines KU812, Lama84, KYO-1, JURL-MK1, EM2, and KLC22, treated with imatinib for 48 h; Imatinib IC-50 for each cell line is depicted (right). **(B)** Table showing upregulated and downregulated NHRs in imatinib sensitive (Control group—KU812, EM2) compared to resistant (Group-1—KCL22, Lama84) CML cell lines based on the RT^2^ NHR profiling (SA Biosciences) results. **(C)** Validation of profile results by qRT-PCR in CML cell lines classified as resistant (JURL-MK1, KCL22, and Lama84) and sensitive (KYO-1, EM2, and KU812) based on imatinib IC-50. The expression of each NHR normalized to *GAPDH*. **(D)** Expression of selected NHRs in granulocytes from healthy donors (*n* = 5) and primary CML bulk cells (*n* = 69).

We performed an *in-vitro* cytotoxicity assay to identify CML cell lines that are sensitive or resistant to IM. KU812, EM2, and KYO1 cell lines were identified as sensitive, and JURL-MK-1, KCL22, and Lama84 cell lines as resistant to IM. The kill curves and IC-50 of the cell lines tested are listed (Figure 1A). To identify whether the NHR expression differs between the IM sensitive (EM2, KU812) and resistant (KCL22 and Lama84) cell lines, we performed NHR profiling using SA Biosciences PCR-based array. There was differential expression (keeping a cut-off ≤ or >2 folds) of 9 NHRs and 13 coregulators between IM-sensitive and resistant cell lines (Figure 1B). Compared to resistant cell lines, 8 NHRs (*ESR1, PPARG, RXRA, RXRB, ESRRG, AHR, AR, THRA*) and ten coregulators (*ITGB3BP, NR2F1, NR1D1, MED17, MED4, NR1H3, NR1I2, NR2F6, NR6A1, MED12*) were upregulated and *RORA* & 4 coregulators (*NR1H4*, *NR0B2*, *NCOA2* & *KAT2B*) were downregulated in imatinib-sensitive cell lines. A Representative Heatmap illustrates the expression profile of NHRs and coregulators (Supplementary Figure S1). Further validation by q-RTPCR revealed NHRs such as *RXRA*, *RXRB,* and *THRA* to be upregulated in the IM-sensitive cell lines compared to IM-resistant cell lines. In contrast, all the other NHRs (*AR*, *ESR1*, *ESRRG*, *PPARG*) were upregulated in at least one of the IM-sensitive cell lines (Figure 1C). To check if the expression pattern of NHRs was unique to CML, we compared the expression of selected NHRs between healthy donor (HD) granulocytes and primary CML cells. There was no significant difference in the expression pattern (Figure 1D).

### 3.2 RXRA ligand treatment significantly reduced IC-50 to imatinib in CML cell lines and primary CML cells

**FIGURE 2:**
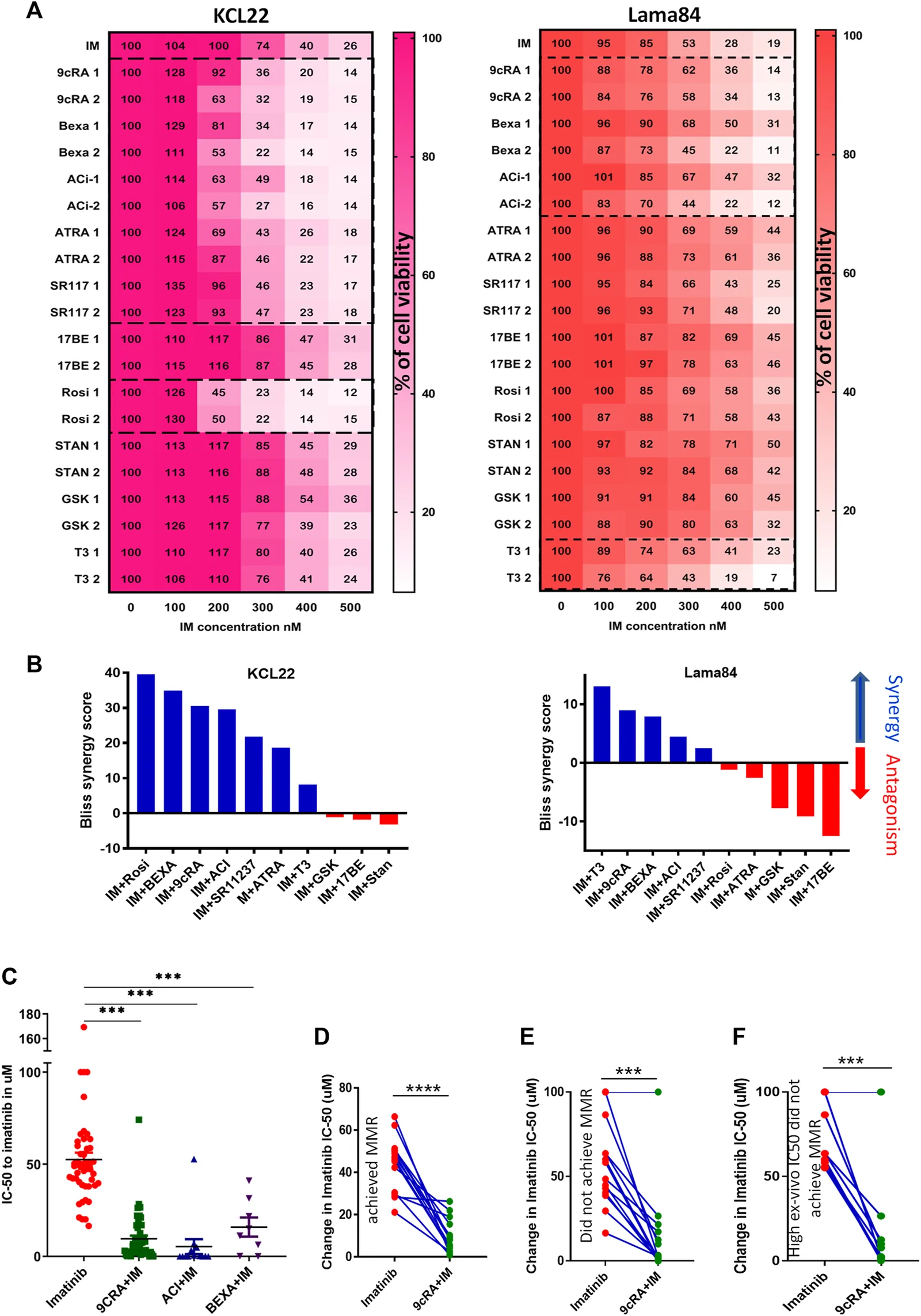
RXRA ligand treatment significantly reduced IC-50 to IM in CML cell lines and primary CML cells **(A)** Representative heat maps showing the cell viability measured at 48 h in CML cell lines KCL22 and Lama84 treated with ligands specific to downregulated NHRs in combination with imatinib. The *X*-axis shows the increasing concentrations of imatinib (100–500 nM), and the *y*-axis shows different NHR ligands (two different concentrations). **(B)** Bliss synergy score were analysed in cell lines treated with NHR ligands in combination with imatinib using synergy finder. The higher the synergy scores, the higher the synergy, and the lower the synergy scores, the antagonism. **(C)** The difference in IC-50 between imatinib alone vs. in combination with ligand [9-cis-retinoic acid (*n* = 42), acitretin (*n* = 13), and bexarotene (n = 8)] was compared using paired *t*-test and the *p*-value calculated by Tukey’s multiple comparison test. The change in imatinib IC-50 with and without 9-cRA in primary CML cells treated with 9-cRA from patients who achieved MMR at 12 months **(D)**, those who did not achieve MMR at 12 months **(E)**, and those who did not achieve MMR with high IC-50 to imatinib **(F)**. *p*-value was calculated by paired *t*-test. Significance values: ***p < 0.001.

We next attempted to see if treatment with ligands specific to the downregulated NHRs in the inherently IM-resistant cell lines (KCL22, Lama84) could improve IM sensitivity. The concentration of the ligands used for this treatment is listed (Supplementary Table S1). Treatment with RXRA ligands (9cRA, bexarotene, acitretin, ATRA, SR11237) and PPARG ligand rosiglitazone decreased cell viability in combination with IM compared to IM alone in the KCL22 cells (Figure 2A). In the Lama84 cells, RXRA ligands bexarotene, acitretin, 9cRA, and THRA ligand T3 decreased cell viability (Figure 2A). The combination index (CI) was calculated using the bliss synergy score mathematical model to test the synergy between the combination of ligand and IM. KCL22 cells treated with rosiglitazone, bexarotene, 9cRA, acitretin, SR11237, ATRA, and T3 showed high synergy with IM, whereas GSK4716, 17-β estradiol, and stanozolol showed antagonism with IM (Figure 2B). Ligands such as T3, 9cRA, bexarotene, acitretin, and SR11237 showed synergy, and rosiglitazone, ATRA, GSK4716, stanozolol and 17-β estradiol showed antagonism with IM in Lama84 cell line (Figure 2B).

Based on these results, the ligands that sensitized either cell line to IM were tested in the primary CML cells. Treatment with RXRA ligands (9cRA, acitretin, and bexarotene) improved IM IC-50 *in-vitro* in primary bulk cells obtained from imatinib naive CML patients (Figure 2C). Other NHR ligands such as ATRA, 17-β-estradiol, pioglitazone, and T3 did not improve IC-50 to IM (Supplementary Figures S2A–E). Based on the patients’ molecular response status at 12 months, the primary CML samples were grouped as attained major molecular response (MMR) or no MMR. 9cRA significantly decreased IM IC-50 *in-vitro* in samples from patients who achieved in all samples irrespective of the molecular response status (Figures 2D, E; Supplementary Figure S3). Primary CML cells from patients who did not achieve MMR and with high IC50 to IM (above median IC-50-53uM) also were sensitized to IM with 9cRA combination treatment (Figure 2F).

### 3.3 RXRA ligand treatment decreased cell viability and colony-forming unit in primary CML CD34^+^ cells

**FIGURE 3:**
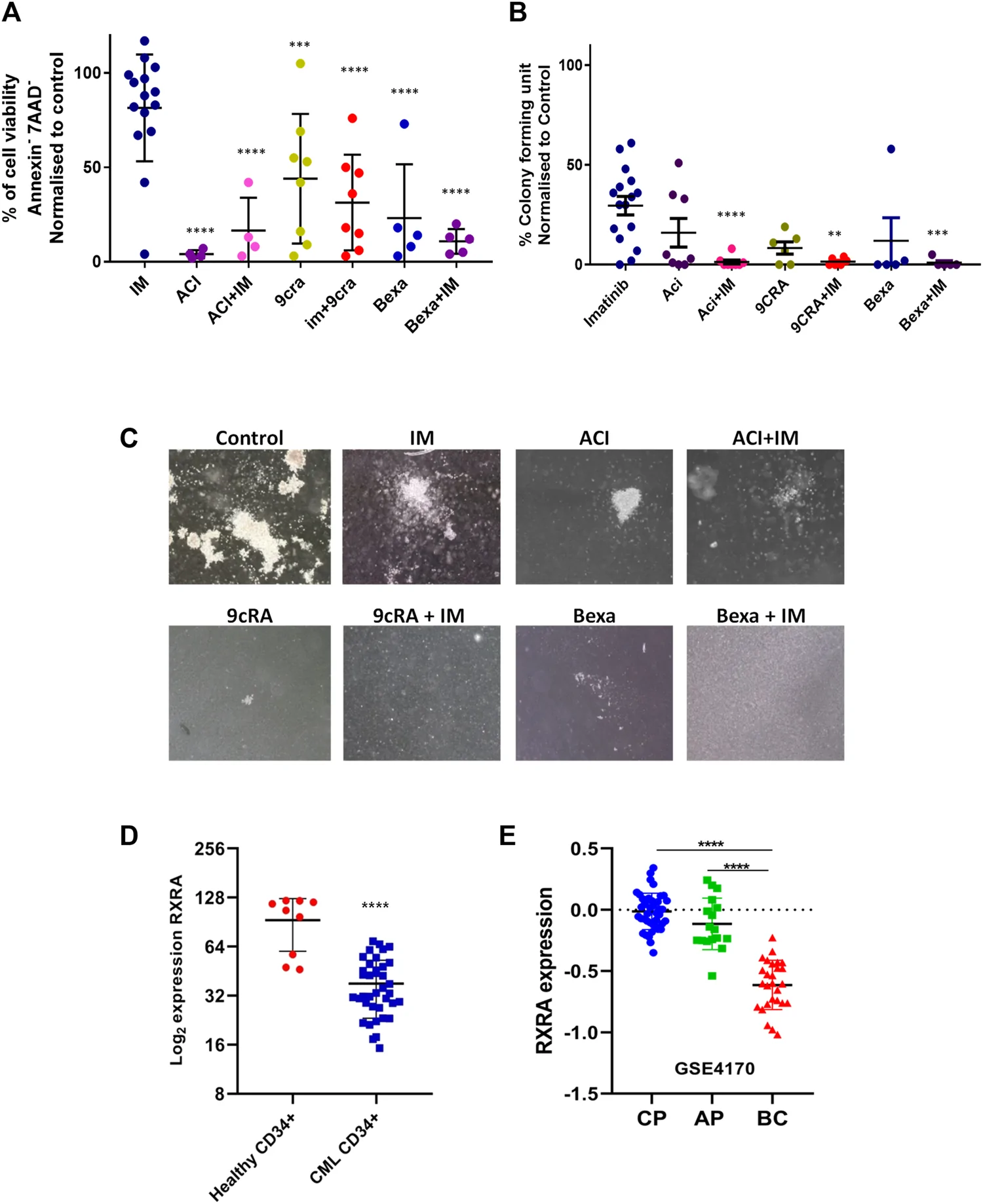
RXRA ligands in combination with imatinib decreases cell viability and colony-forming unit in primary CML CD34^+^ cells **(A)** Percentage cell viability in primary CML CD34^+^ cells treated with rexinoids (Aci *n* = 4, 9CRA *n* = 8 & Bexa *n* = 5) in combination with imatinib. Significance was calculated compared to imatinib-treated cells. *p*-value was calculated by Tukey’s multiple comparison test. **(B)** Colony-forming capacity in primary CML CD34^+^ cells treated with rexinoids (Aci *n* = 8, 9CRA *n* = 6 & Bexa *n* = 5) combined with imatinib; colonies were scored on day 14. Significance was calculated compared to imatinib-treated cells. *p*-value was calculated by Tukey’s multiple comparison test. **(C)** Representative images of the colony-forming units in primary CML CD34^+^ cells treated with imatinib alone, rexinoids alone, and in combination. **(D)** *RXRA* mRNA expression in healthy donor CD34^+^ cells (*n* = 9) and CML CD34^+^ (*n* = 39) cells analyzed using Nanostring nCounter. *RXRA* expression was normalized to *ACTB, GAPDH & GUSB* and presented in Log2. **(E)** RXRA expression values from the GSE4170 dataset in CML chronic phase (CP), accelerated phase (AP), and blast crisis (BC) patients. *p*-value was calculated by the Mann-Whitney *U* test. Significance values: **p < 0.01; ***p < 0.001.

As IM treatment does not eliminate the CML LSCs, we next tested the effect of RXRA ligands alone and in combination with IM in purified primary CML CD34^+^ cells *in-vitro*, and the cell viability and colony-forming capacity were assessed. RXRA ligands, either alone or combined with IM, significantly decreased cell viability (Figure 3A) and colony-forming capacity (Figures 3B, C)*.* Interestingly, acitretin alone treated CML CD34^+^ cells showed a significant reduction in cell viability compared to imatinib alone, 9cRA alone, or Bexa alone or in combination (Figure 3A). Treatment of healthy PBMNCs with RXRA ligands 9cRA, bexarotene, and acitretin in combination with IM did not reduce cell viability compared to IM alone, suggesting that the cytotoxic effect of RXRA ligands in combination with imatinib is specific to CML cells (Supplementary Figures S4A–C). We then compared the expression of *RXRA* in CML CD34^+^ and HD CD34^+^ cells. *RXRA* expression was significantly lower in CML CD34^+^ cells than in HD CD34^+^ cells (Figure 3D). We also probed for *RXRA* expression across CML chronic phase (CP), accelerated phase (AP), and blast crisis (BC) patient samples from the GSE4170 dataset and found significantly reduced expression of *RXRA* in BC-CML samples compared to CP and AP (Figure 3E). These results suggest that either alone or combined with IM, RXRA ligands could selectively target CML CD34^+^ cells.

### 3.4 RXRA ligand treatment inhibits BCR-ABL downstream signaling and activates the apoptotic cascade in CML cells in combination with imatinib

**FIGURE 4:**
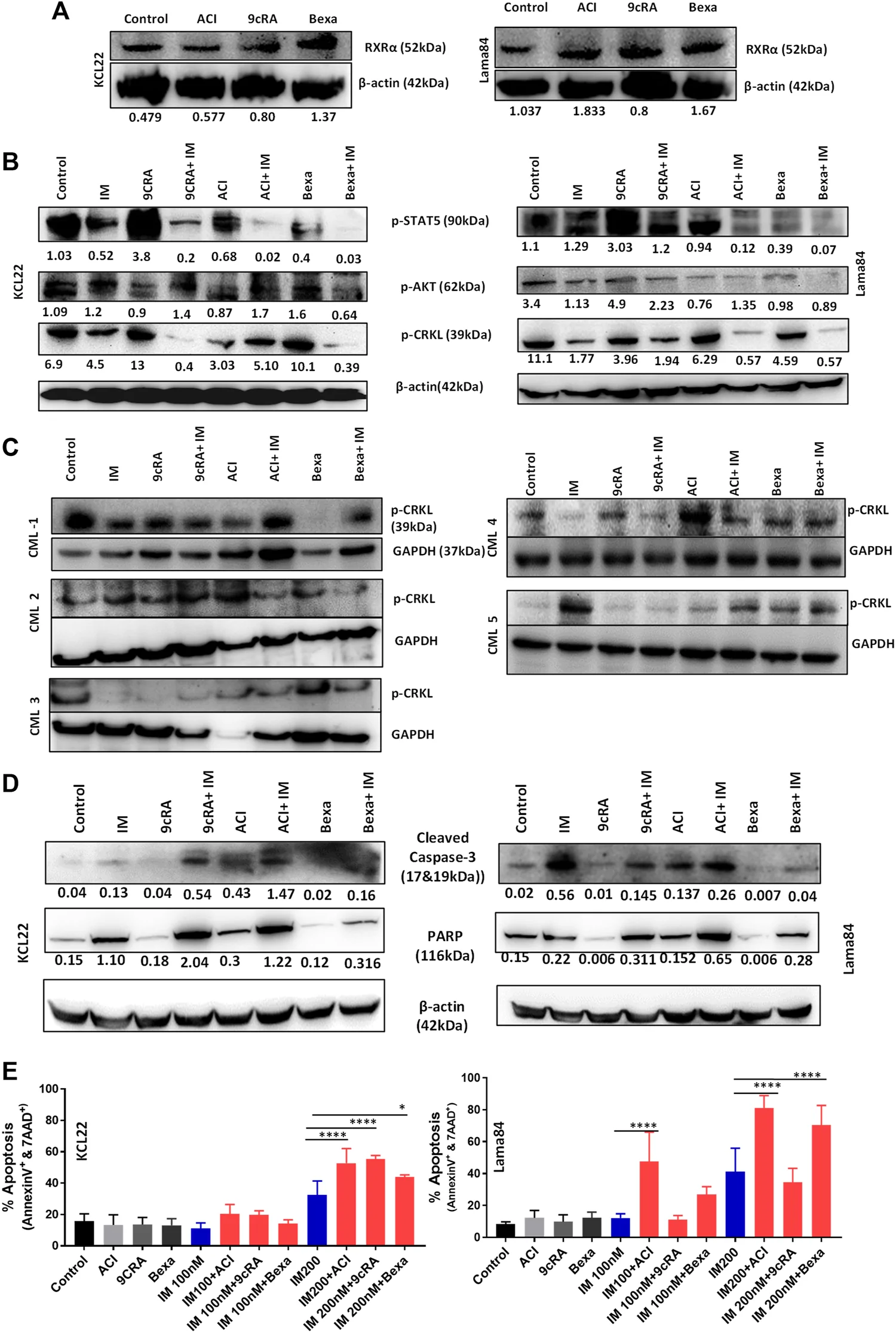
RXRA ligands decrease BCR-ABL signaling and activate the apoptosis pathway in CML cell lines **(A)** Western blot image showing increased RXRA protein expression in Lama84 and KCL22 cell lines treated with RXRA ligands Aci, 9CRA, and Bexa. β-actin was used as the loading control. **(B)** Western blot image showing the expression of BCR-ABL downstream signaling pathway proteins p-CRKL, p-AKT & p-STAT5 in KCL22, Lama84 cell lines, and **(C)** primary CML cells (*n* = 5) treated with rexinoids for 24 h, followed by imatinib treatment. β-actin/GAPDH was used as a loading control. **(D)** Western blot image showing the expression of apoptotic proteins of cleaved caspase-3 & PARP in CML cell lines treated with rexinoids followed by imatinib; β-actin was used as a loading control. **(E)** The percentage of apoptosis (Annexin-V and 7AAD positive cells) in CML cell lines treated with rexinoids with or without imatinib. *p*-value calculated by Tukey’s multiple comparison test.

RXRA forms a homodimer or heterodimerizes with other NHRs, such as RARA, VDR, and PPARG to regulate cellular proliferation, differentiation, and apoptosis (Gilardi and Desvergne, 2014). To identify the expression of other NHRs post RXRA ligand treatment in CML primary cells, RNA expression of *RARA*, *VDR,* and *PPARG* was tested. RXRA ligands treatment resulted in consistent upregulation of *RARA* and *VDR* and downregulation of *PPARG* (Supplementary Figure S5). RXRA ligand treatment also increased RXRA protein levels in the CML cell lines (Figure 4A).

We then checked the effect of these RXRA ligands on the BCR-ABL signaling pathways (p-CRKL as a marker of proliferation, p-AKT as a marker of survival, and p-STAT5 as a marker of quiescence) in CML cell lines and primary cells. RXRA ligands decreased p-CRKL, p-AKT, and p-STAT5 at the protein level in combination with IM (Figure 4B) in CML cell lines. Primary CML cells treated with RXRA ligands combined with IM also showed inhibition of downstream signaling pathway p-CRKL (Figure 4C).

Next, we examined the effect of these RXRA ligands on CML cells in inducing apoptosis. CML cell lines treated with RXRA ligands combined with IM showed a significant increase in cleaved caspase-3 and cleaved PARP expression (Figure 4D) and increased apoptosis combined with IM (Figure 4E) as well as with 2^nd^ generation TKIs, dasatinib and nilotinib (Supplementary Figures S6A, B). These results indicate that RXRA ligands improve IM sensitivity by decreasing the BCR-ABL downstream signaling pathways and increasing apoptosis in CML primary cells and cell lines.

### 3.5 Molecular overexpression of *RXRA* in Imatinib resistant Lama84 cell line decreased proliferation, BCR-ABL downstream signaling, and OXPHOS resulting in improved imatinib sensitivity

**FIGURE 5:**
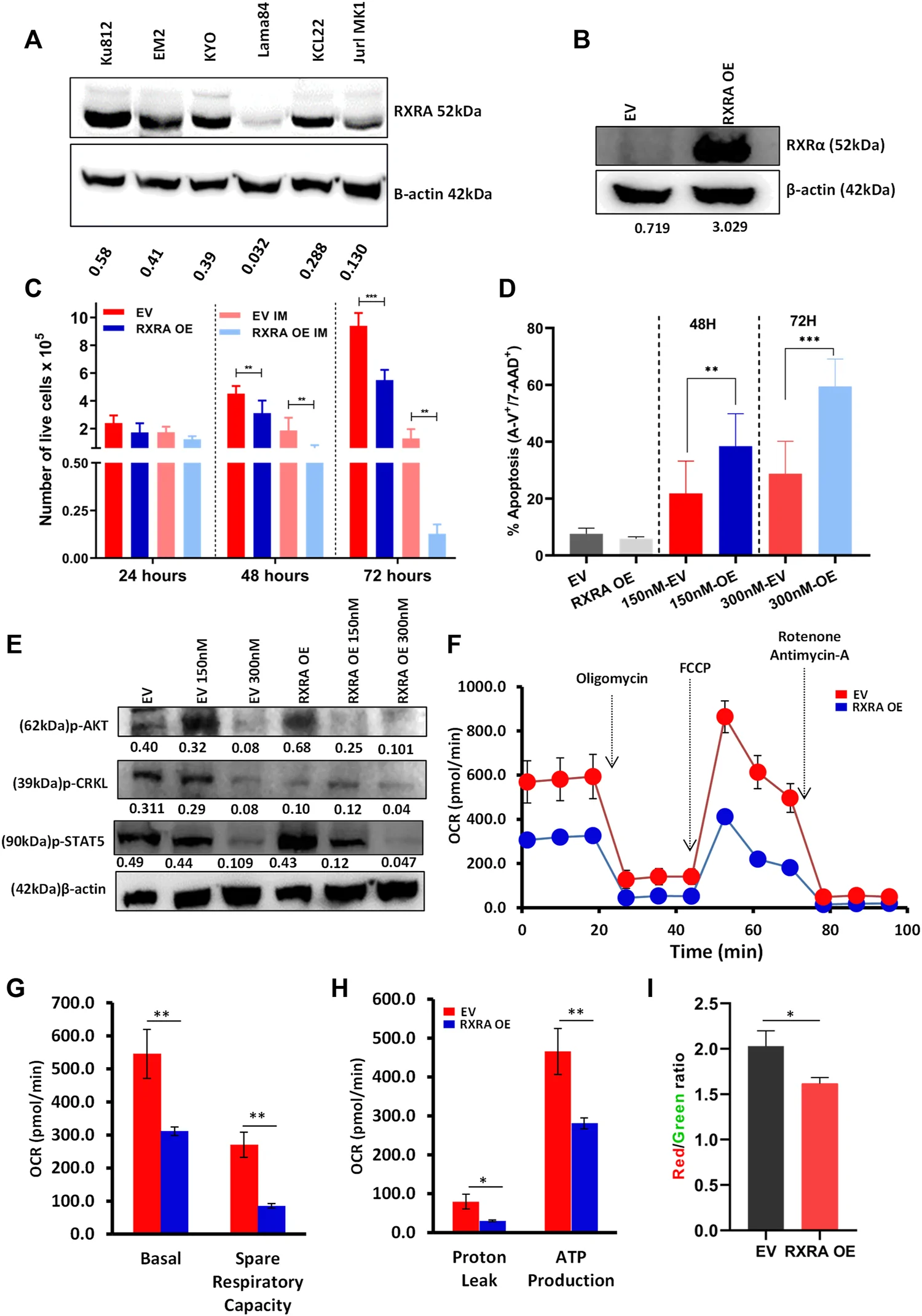
Molecular overexpression of RXRA improves imatinib sensitivity in the Lama84 cell line by inhibiting BCR-ABL signaling and oxidative phosphorylation **(A)** Basal RXRA expression in whole-cell lysate from CML cell lines KU812, EM2, Lama84, and KCL22 analyzed by western blot. Lama84 cell line with relatively low RXRA protein expression was transduced with RXRA OE plasmid, and overexpression was confirmed at the protein levels by western blotting **(B)**. The number of viable cells in Lama84 RXRA OE vs. EV with and without imatinib treatment was assessed using trypan blue exclusion assay (*n* = 3) at three different time points. The doubling time was calculated using exponential curve analysis, and the *p*-value was calculated using the Mann-Whitney *U* test **(C)**. EV and RXRA OE Lama84 cells were treated with 150 nM concentration of imatinib (*n* = 3), and the percentage of apoptosis was assessed by apoptosis assay (Annexin-V and 7AAD positive cells) at two different time points **(D)**. The *p*-value was calculated by Tukey’s multiple comparison test. Western blot image showing the expression of BCR-ABL downstream signaling proteins (p-CRKL, p-AKT, and p-STAT5) in RXRA OE cells and EV cells **(E)**. Mito Stress Test for EV and RXRA OE cells using Sea horse extracellular flux analyzer **(F)**. Quantitative assessment of Basal OCR rates, Spare respiratory capacity, proton leak, and ATP production **(G,H)** (*N* = 4). **(I)** The quantitavie measure of mitochondrial membrane potential was analysed using the ratio of JC-1 dimer (Red) by JC-1 monomer (Green).

As proof of principle, we evaluated if molecular overexpression of *RXRA* in the CML cell line would result in similar effects as that of ligand treatment. Overexpression (OE) of *RXRA* in the Lama84 cells with deficient basal protein expression (Figure 5A) of RXRA, resulted in significantly increased RXRA expression at the protein level (Figure 5B). Interestingly, RXRA OE alone considerably decreased the proliferative capacity of these cells and increased the sensitivity to imatinib in a time-dependent manner (Figure 5C; Supplementary Figure S7). We further validated the sensitivity to imatinib in RXRA OE cells by apoptosis and observed enhanced cytotoxicity to imatinib in these cells compared to the empty vector (EV) transduced (Figure 5D). We next assessed if the profound sensitivity to imatinib is due to inhibition of BCR-ABL downstream signaling pathways (p-CRKL, p-AKT & p-STAT5) as observed during ligand treatment. Immunoblotting of RXRA OE and EV cells revealed diminished p-CRKL signaling in the RXRA OE cells at the basal level. We observed complete inhibition of p-STAT5, p-CRKL, and p-AKT in a dose-dependent manner compared to EV, post-IM treatment in the RXRA OE cells (Figure 5E).

As leukemic cells rely on oxidative phosphorylation for energy demand and for withstanding TKI therapy, we assessed the bioenergetic profile of EV and RXRA OE cells. Intriguingly, RXRA OE markedly reduced the basal respiration rates indicative of restricted intrinsic ATP demand. RXRA OE significantly reduced ATP production, and the addition of mitochondrial uncoupler FCCP showed markedly reduced oxidative bursts compared to EV (Figures 5F–H). We further assessed the mitochondrial membrane potential (MMP) of EV and RXRA OE using JC-1 and found significantly reduced MMP in RXRA OE cells (Figure 5I). Interestingly, MMP measured post-RXRA ligand treatment in CML cell lines showed a significant decrease in MMP in the ACI-treated KCL22 cells, while there was no substantial change in Lama84 cells (Supplementary Figures S8A, B). These results collectively suggest that RXRA OE mimics the effect of RXRA ligand treatment in CML cells. Additionally, molecular and pharmacological activation of RXRA could potentially inhibit OXPHOS in CML cells improving sensitivity to IM.

### 3.6 RXRA ligand treatment and RXRA OE decreased leukemic burden and improved survival in cell-derived xenograft CML mice model by enhancing sensitivity to imatinib

**FIGURE 6:**
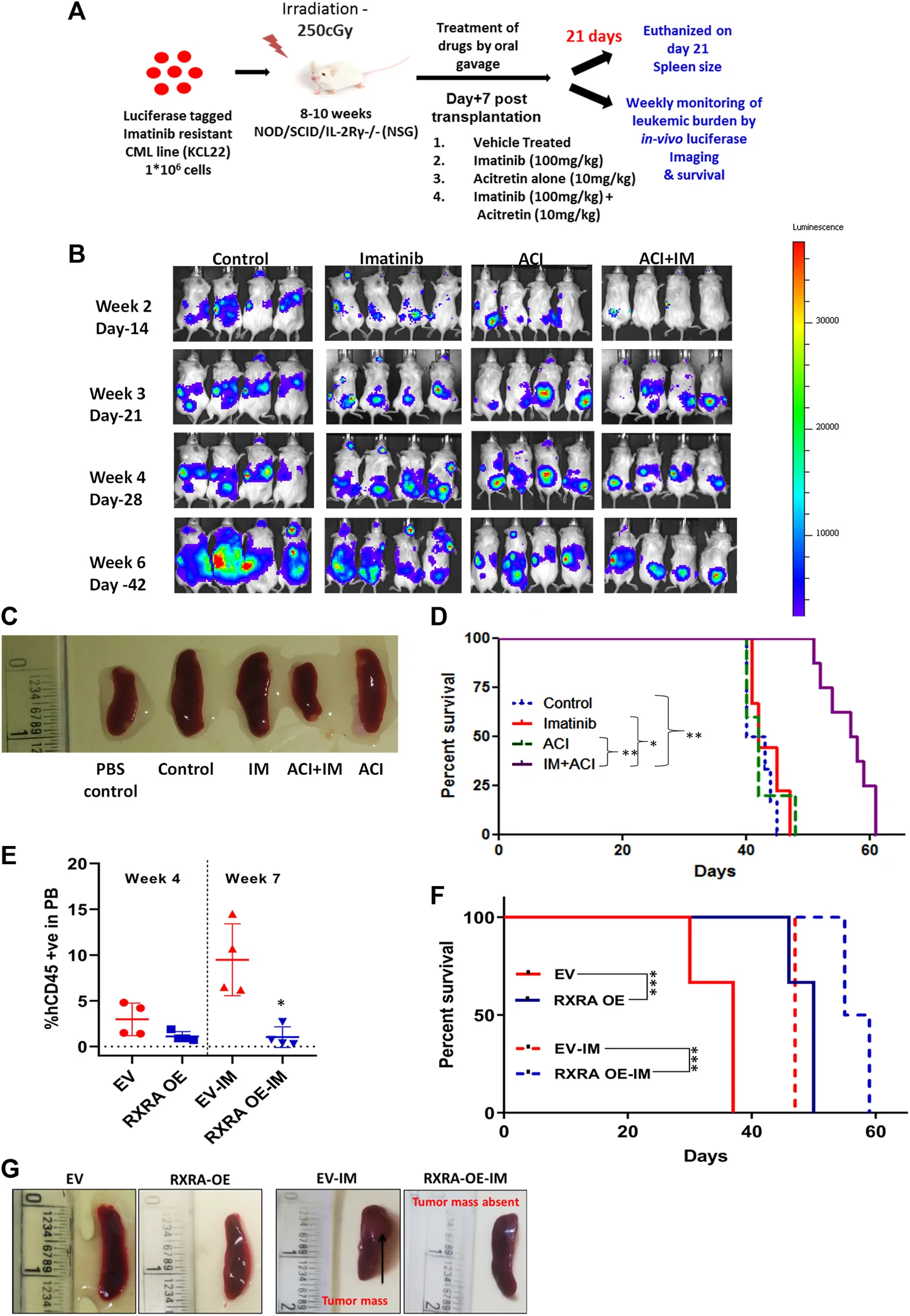
RXRA ligand acitretin in combination with imatinib decreases the leukemic burden and increases survival in the xenograft CML mice model **(A)** Outline of the CML cell line-derived (CDX) mouse model experiment. **(B)** Representative bioluminescence images showing *in-vivo* luciferase expression in CML mice treated with RXRA ligand with or without imatinib compared to the vehicle control mice. **(C)** Representative mice spleens in vehicle vs. treated animals. **(D)** Kaplan-Meier survival curve of NSG mice transplanted with KCL22 cell line and treated with IM alone (*n* = 9), ACI alone (*n* = 5), ACI + IM (*n* = 8), and vehicle control (*n* = 6). Percentage survival was calculated using the Kaplan-Meier analysis, and median survival was compared between treated groups vs. control groups by Dunn’s multiple comparisons test. **(E)** Percentage of human CD45 cells from EV and RXRA OE injected mice after 4 weeks of transplantation and at week 7 between EV/RXRA OE treated with IM. **(F)** Kaplan-Meier survival curve of NSG mice transplanted with Lama84 EV (*n* = 3) and RXRA OE cell line (*n* = 4) (2*10^6^ cells transplanted/mouse). Percentage survival was calculated using the Kaplan-Meier analysis, and median survival was compared between treated groups vs. control groups by Dunn’s multiple comparisons test. **(G)** Representative spleens from mice injected with wild type vs. RXRA OE Lama84 cell line.

As the *in-vitro* data suggested that RXRA ligands improved imatinib sensitivity and inhibited BCR-ABL downstream signaling in CML cell lines and primary CML cells, we investigated the *in-vivo* efficacy of acitretin combined with imatinib. A transplantable xenograft CML mouse model was developed by injecting luciferase-expressing KCL22 cells into sub-lethally irradiated NSG mice. Mice were treated with imatinib, acitretin, or a combination of both for 21 days (Figure 6A). Acitretin-treated mice showed ruffled fur coat and moderate weight loss. Intriguingly, mice treated with either imatinib alone or acitretin alone showed an increased incidence of hind leg paralysis compared to vehicle-treated mice and did not improve survival. While the combination of acitretin and IM treatment reduced spleen size, decreased leukemic burden, and significantly increased median survival (Figures 6B–D). *In-vitro* treatment of the HD PBMNCs and CD34^+^ cells with acitretin in combination with imatinib did not significantly affect the viability of these cells as measured by apoptosis assay, suggesting that this effect is unique to CML cells (Supplementary Figures S9A, B).

Next, we evaluated the engraftment capacity of the RXRA OE and EV cells by transplanting an equal number of cells into sub-lethally irradiated NSG mice (Supplementary Figure S9C). RXRA OE hindered the engraftment capacity of the Lama84 cell line and improved survival. We also assessed the sensitivity to imatinib *in-vivo* in the RXRA OE/EV engrafted mice. Imatinib treatment for 21 days significantly reduced leukemic burden and prolonged survival in the RXRA OE cells transplanted mice compared to EV (Figures 6E, F; Supplementary Figure S9D). Imatinib treatment in EV-transplanted mice showed tumor mass formation but was absent in the RXRA OE mice treated with imatinib (Figure 6G). These comprehensive *in-vivo* results suggest that pharmacological and molecular approaches to activate RXRA could hinder leukemic cell engraftment, increase sensitivity to imatinib, and improve survival.

## 4 Discussion

Suboptimal response to TKI or TKI failure is associated with poor progression-free survival in patients with CML. Combining TKI with small molecules specific to pathways in which imatinib-resistant cells escape therapy could help eliminate the residual LSCs. In the present study, we observed differential expression of NHRs in imatinib sensitive vs. resistant CML cell lines when comprehensively screened for the expression of NHR and coregulators across CML cell lines. Upon treatment with ligands specific to the downregulated NHRs in the imatinib-resistant CML cell lines, only RXRA ligand treatment showed improved sensitivity to IM in both cell lines. Pioglitazone, a PPARG ligand, has been reported to eliminate CML LSCs but not the bulk primary CML cells (Prost et al., 2015; Wang et al., 2017). Combination treatment with Pioglitazone and imatinib induced complete molecular response (CMR) in patients who have not achieved CMR under long-term imatinib treatment in CML patients and remained in CMR after the withdrawal of imatinib (Prost et al., 2015).

Treating with ligands specific to the downregulated NHRs, including rosiglitazone (a PPARG ligand), showed a differential effect, sensitizing the KCL22 cell line but no impact on the Lama84 cell line in combination with IM. Similarly, THRA agonist T3 treatment sensitized Lama84 and did not affect KCl22. Although 17BE and stanozolol treatment improved viability in both cell lines, these ligands did not improve sensitivity to IM in bulk CML cells *ex-vivo*. As these ligands are present endogenously, it would be interesting to understand their effect on primary CML cells. Hence, we worked with RXRA ligands 9cRA, Aci, and bexarotene, which improved IM sensitivity in CML bulk and CML CD34^+^ cells but had minimal effect on HD CD34^+^ cells and PBMNCs. In addition, treatment with RXRA ligands combined with imatinib decreased clonogenic potential in primary CML CD34^+^ cells.

Prost et al. (2015) showed inhibition of p-STAT5 led to clearance of CML LSCs after treatment with *PPARG* agonist pioglitazone. When we assessed the BCR-ABL downstream signaling pathways, we observed that RXRA agonist in combination with IM resulted in decreased p-CRKL, p-STAT5, and p-AKT along with activation of the apoptotic cascade in CML cell lines and primary cells.

Lentiviral-mediated overexpression of *RXRA* in imatinib-resistant Lama84 cell line showed decreased proliferation, decreased BCR-ABL downstream signaling, and improved imatinib sensitivity *in-vitro*. Di Martino et al. (2021a) showed that deleting endogenous RXRA in an MLL-AF9 AML mouse model increased leukemogenic potential and reduced survival. The same authors subsequently reported that a constitutively overexpressing mutant variant of RXRA resulted in myeloid maturation and prolonged survival in the murine model (Di Martino et al., 2021a; Di Martino et al., 2021b). In line with this, in our RXRA overexpressing CDX CML mice model, we observed decreased leukemic burden and improved survival compared to EV-transduced CDX. We also found a significant reduction in *RXRA* expression in CML CD34^+^ cells compared to HD CD34^+^ and a reduction in the expression of *RXRA* in blast crisis CML patients compared to chronic phase, accelerated phase CML from the GSE4170 dataset indicating a potential role of *RXRA* in hindering leukemogenic capacity in CML.

It is reported that CML CD34^+^ cells have a high oxidative capacity, which could be inhibited using tigecycline (Kuntz et al., 2017). Interestingly, we also identified that RXRA OE markedly inhibited the OXPHOS capacity of CML cells. Potentially RXRA ligands could mimic RXRA OE and have a disruptive effect on the oxidative potential of CML cells. In addition, RXRA OE and imatinib treatment significantly reduced leukemic cell growth *in-vivo* resulting in prolonged survival. Studies in solid tumors have demonstrated using RXRA agonists to increase sensitivity to chemotherapeutic drugs cisplatin and paclitaxel (Hermann et al., 2005; Blumenschein et al., 2008; Ramlau et al., 2008). Similar results were observed when the CML CDX model (KCL22 cell line) was treated with RXRA agonist acitretin combined with IM, improving survival. Interestingly, the agonist-treated arm had no survival benefit, unlike RXRA OE. The mechanisms of RXRA activation by pharmacological/molecular means may differ, and RXRA agonists (9cRA, ACI, and BEXA) may have a pleiotropic effect *in-vivo* compared to RXRA OE.

While our findings suggest the potential use of RXRA agonists in combination with IM in eliminating CML CD34^+^ *in-vitro* and sensitizing CML bulk cells highly resistant to IM (IC50 > 53uM), a more robust *in-vivo* model to evaluate the therapeutic efficacy of this combination and anti-leukemogenic potential RXRA has on CML LSCs is needed. We are currently exploring the mechanism by which RXRA activation by both molecular/pharmacological means could orchestrate the downstream kinase activities.

We demonstrated differential NHR expression between imatinib-resistant and sensitive cell lines. The ligand-dependent activation of RXRA could be an effective CML target, affecting BCR-ABL1 downstream signaling and apoptotic pathway. Our *in-vitro* and *in-vivo* results suggest that using RXRA ligands could be an effective combination therapy to improve the imatinib response in patients with CML.

## Data Availability

The original contributions presented in the study are included in the article/Supplementary Material, further inquiries can be directed to the corresponding author.
