# Correction: Modulating retinoid-X-receptor alpha (RXRA) expression sensitizes chronic myeloid leukemia cells to imatinib *in vitro* and reduces disease burden *in vivo*

**DOI:** 10.3389/fphar.2026.2006729

**Published:** 2026-10-07

**Authors:** Bharathi M. Rajamani, Raveen Stephen Stallon Illangeswaran, Esther Sathya Bama Benjamin, Balaji Balakrishnan, Daniel Zechariah Paul Jebanesan, Saswati Das, Aswin Anand Pai, Rakhi Thalayattu Vidhyadharan, Ajith Mohan, Sreeja Karathedath, Aby Abraham, Vikram Mathews, Shaji R. Velayudhan, Poonkuzhali Balasubramanian

**Affiliations:** 1 Department of Haematology, Christian Medical College, Vellore, India; 2 Department of Biotechnology, Thiruvalluvar University, Vellore, India; 3 Sree Chitra Tirunal Institute for Medical Sciences and Technology, Thiruvananthapuram, India; 4 Department of Integrative Biology, School of Bio Sciences and Technology, Vellore Institute of Technology, Vellore, India; 5 Manipal Academy of Higher Education, Manipal, India; 6 Centre for Stem Cell Research (CSCR), A Unit of InStem Bengaluru, Christian Medical College Campus, Vellore, India

**Keywords:** chronic myeloid leukemia (CML), imatinib, resistance, RXRA, RXRA ligands

In the published article, there was an error in Materials and Methods, section 2.11 “Overexpression of RXRA in CML cell line”. The source of the RXRA plasmid was stated incorrectly. The plasmid was described as “pLX304 (clone ID- HsCD00437134)” purchased from the DNASU plasmid repository (Arizona, United States); the correct plasmid is TFORF2199, obtained from Addgene (Addgene plasmid #141967).

A correction has been made to Materials and Methods, 2.11 Overexpression of RXRA in CML cell line:

“The RXRA plasmid containing the full-length cDNA (TFORF2199) was purchased from Addgene (Addgene plasmid #141967; RRID: Addgene_141967). Lentivirus particles were prepared by transfecting 293T cells with the RXRA cDNA plasmid and packaging plasmids. Lentiviruses were collected after 48, 60, and 72 h, pooled together, filtered and concentrated using a lenti-X concentrator (Takara, Bio, Shiga, Japan), and stored at −80 °C until use. Lama84 cells were transduced with lentiviruses in the presence of polybrene, followed by spinfection. RXRA OE virus transduced Lama84 cells were selected by puromycin (1 μg/mL).”

The original version of this article has been updated.

