# Supplementary material for: Modulating retinoid-X-receptor alpha (RXRA) expression sensitizes chronic myeloid leukemia cells to imatinib in vitro and reduces disease burden in vivo
Source: Front Pharmacol. 2023 May 31;14:1187066. doi: 10.3389/fphar.2023.1187066 (PMC10264673; doi:10.3389/fphar.2023.1187066)
Supplement: Supplementary file 1 [file Presentation1.PPTX]

## Slide 1
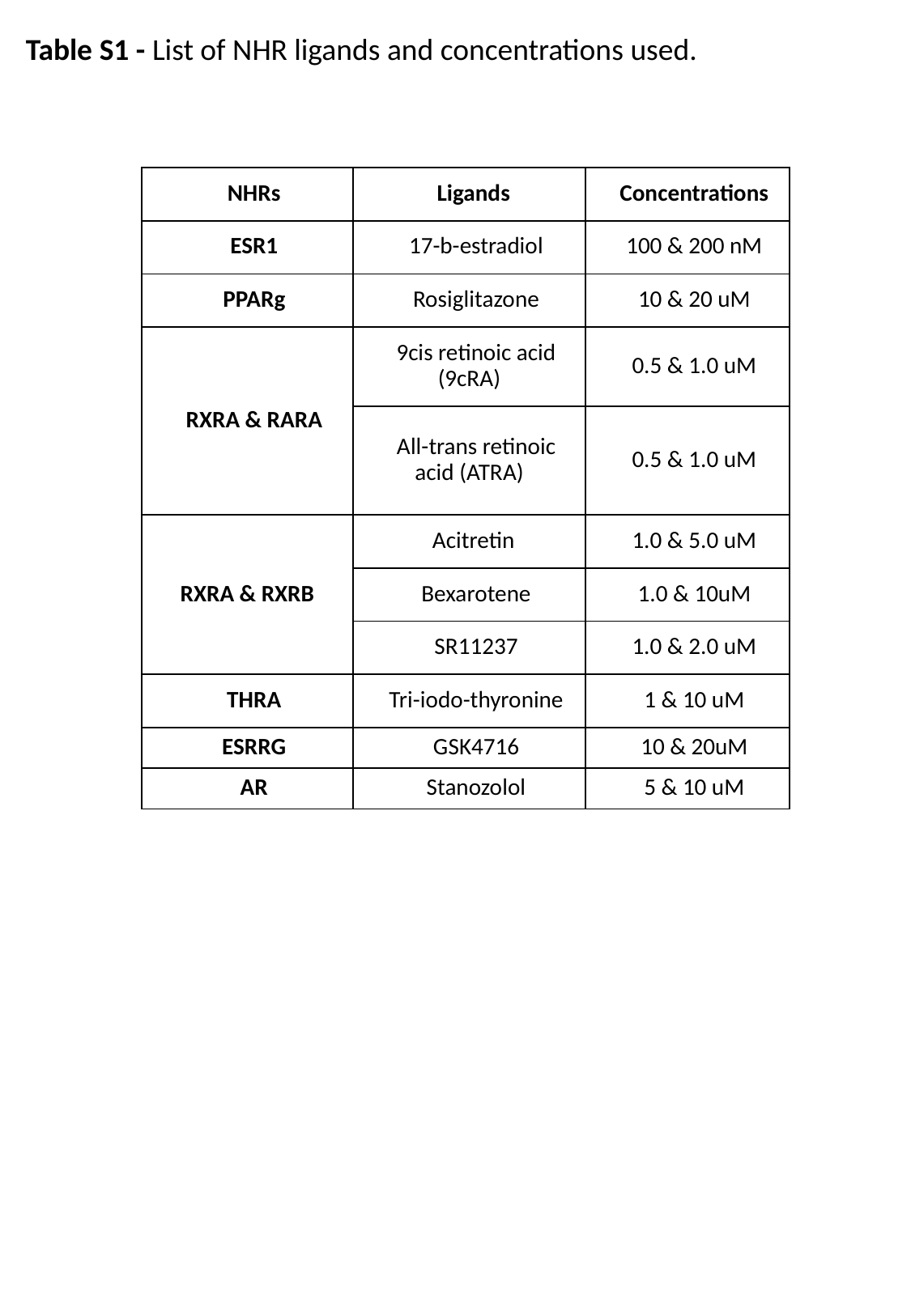

Table S1 - List of NHR ligands and concentrations used.
| NHRs | Ligands | Concentrations |
| --- | --- | --- |
| ESR1 | 17-b-estradiol | 100 & 200 nM |
| PPARg | Rosiglitazone | 10 & 20 uM |
| RXRA & RARA | 9cis retinoic acid (9cRA) | 0.5 & 1.0 uM |
| | All-trans retinoic acid (ATRA) | 0.5 & 1.0 uM |
| RXRA & RXRB | Acitretin | 1.0 & 5.0 uM |
| | Bexarotene | 1.0 & 10uM |
| | SR11237 | 1.0 & 2.0 uM |
| THRA | Tri-iodo-thyronine | 1 & 10 uM |
| ESRRG | GSK4716 | 10 & 20uM |
| AR | Stanozolol | 5 & 10 uM |

## Slide 2
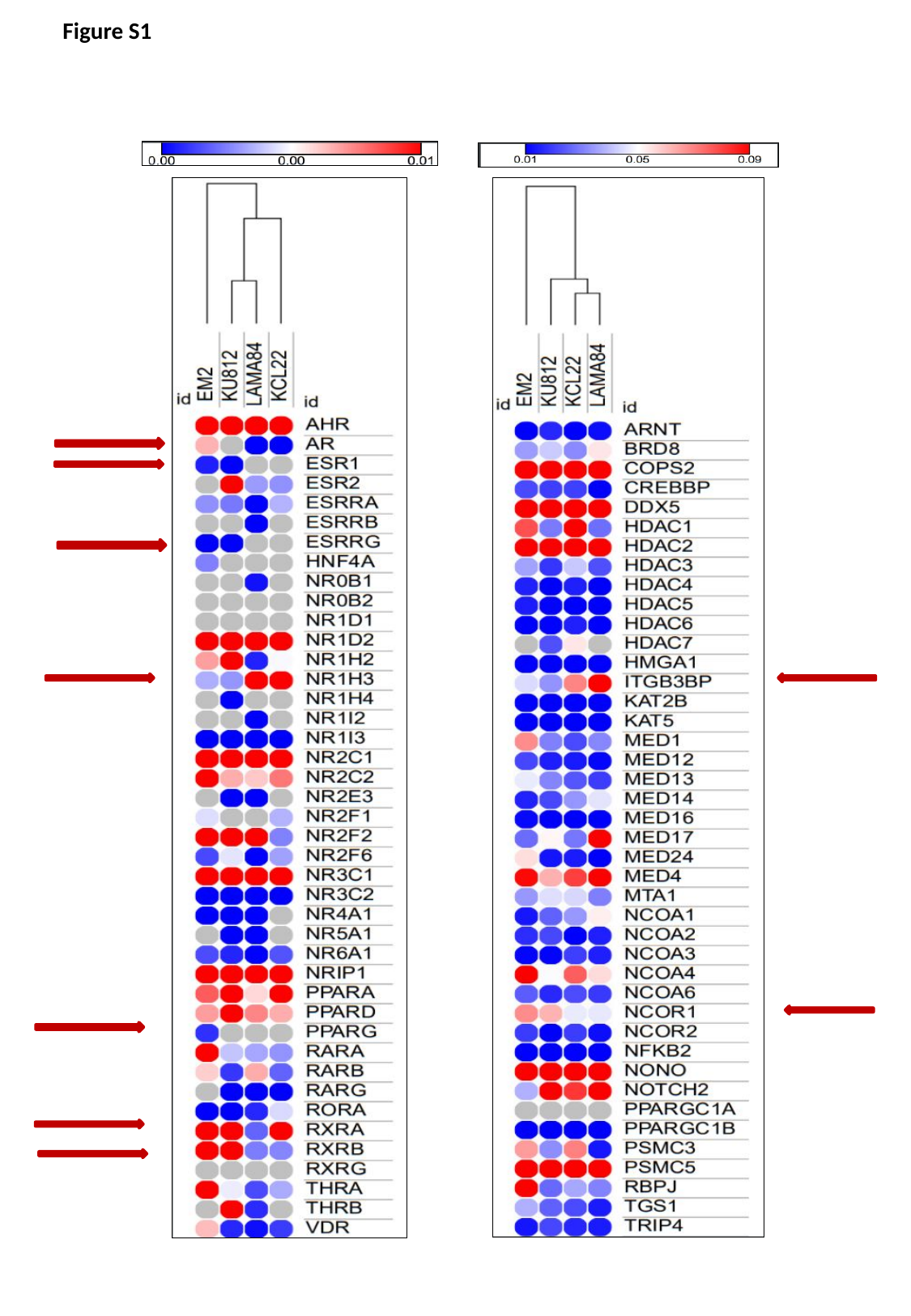

Figure S1

## Slide 3
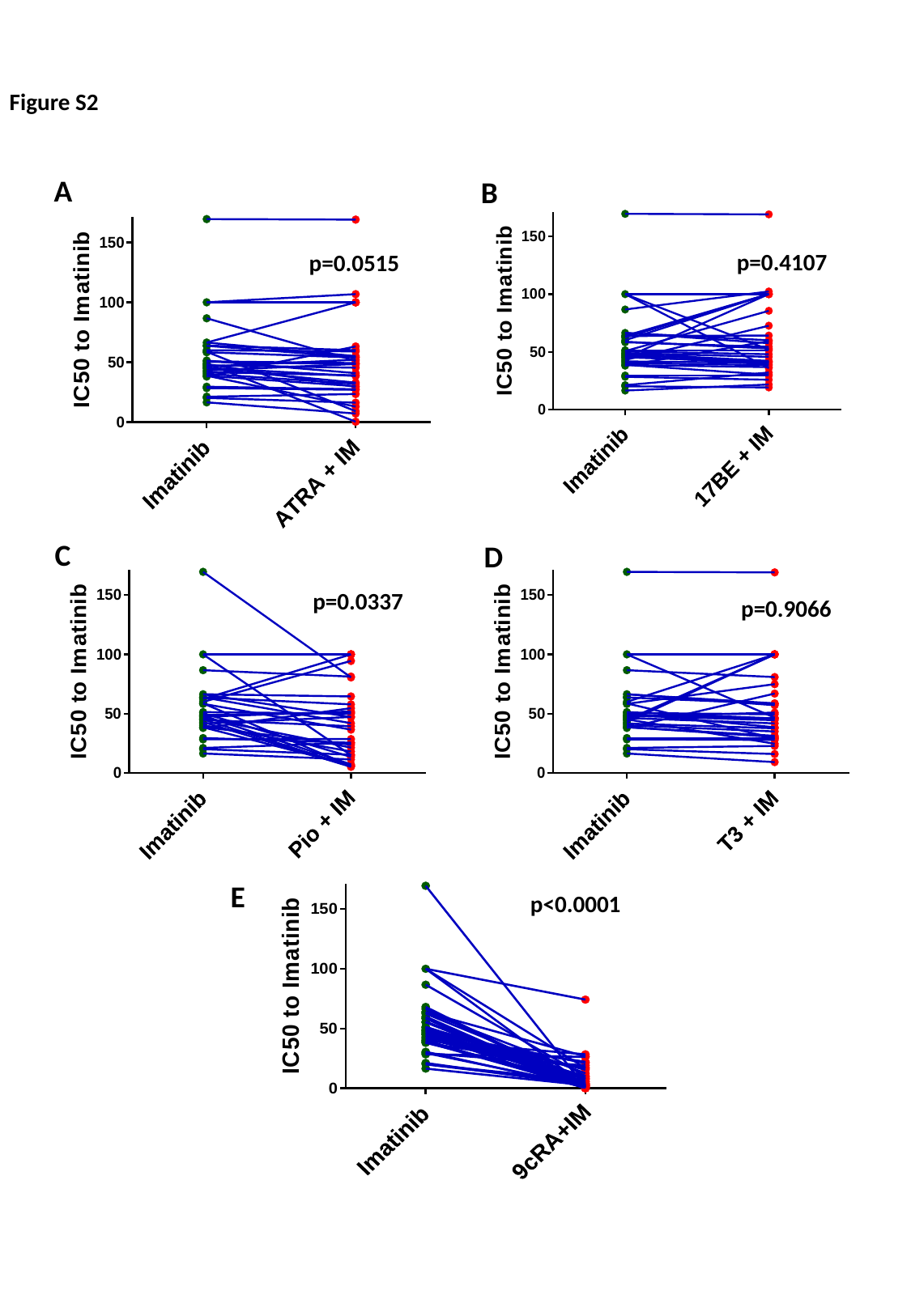

Figure S2
A
B
p=0.4107
p=0.0515
C
D
p=0.0337
p=0.9066
p<0.0001
E

## Slide 4
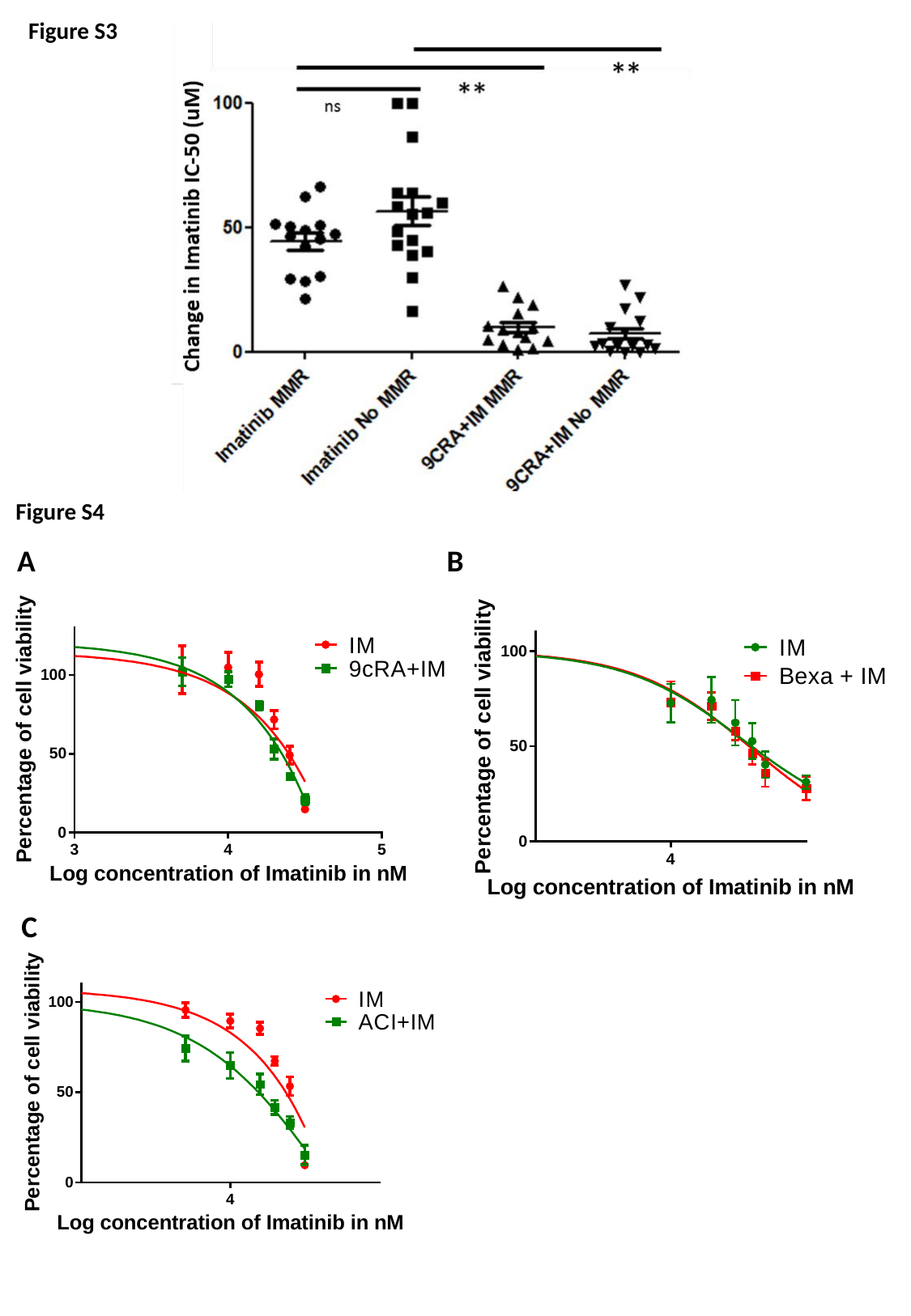

Figure S3
Figure S4
A
B
C

## Slide 5
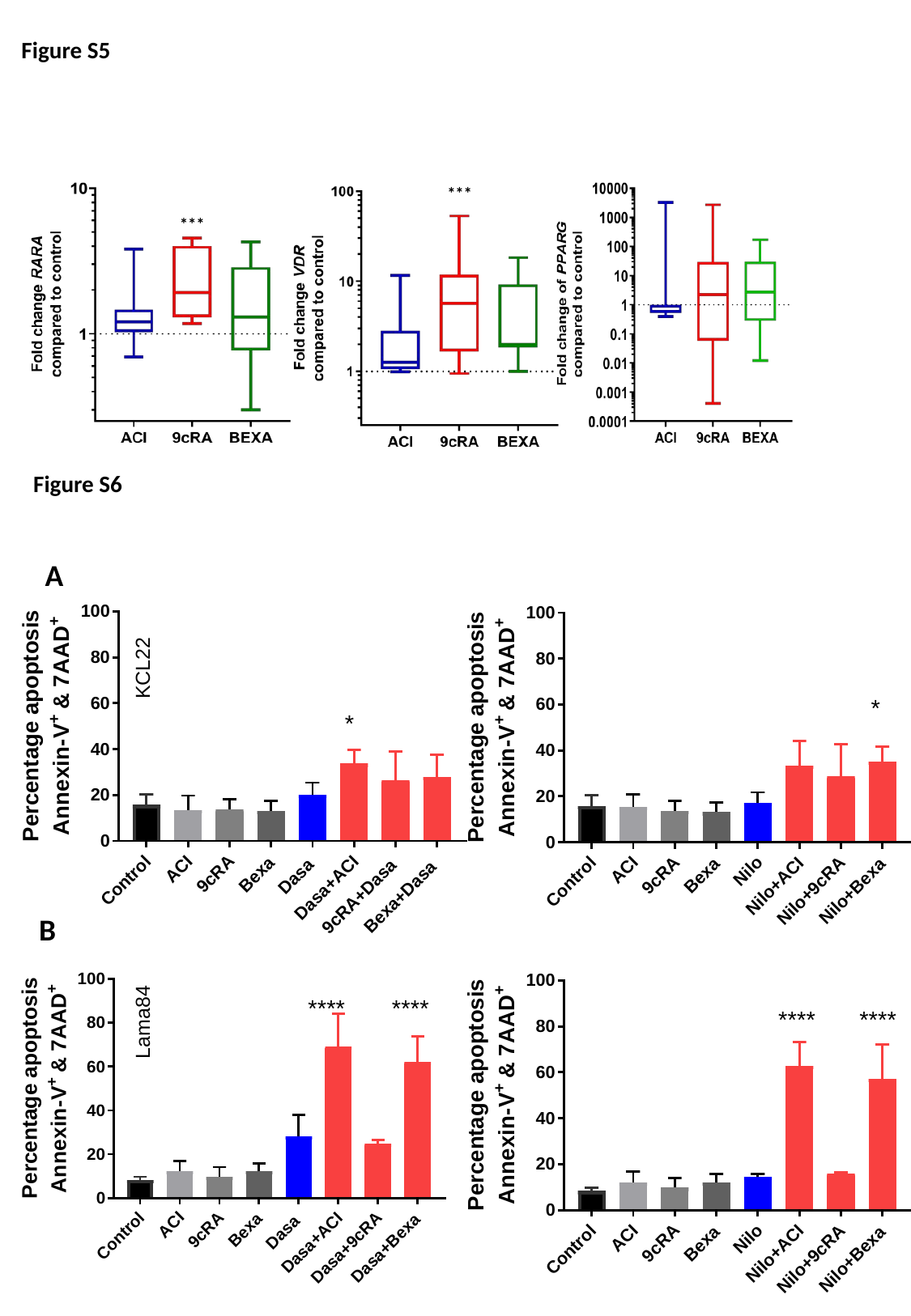

Figure S5
***
***
Figure S6
A
KCL22
| \* |
| --- |
| \* |
| --- |
B
| \*\*\*\* |
| --- |
| \*\*\*\* |
| --- |
Lama84
| \*\*\*\* |
| --- |
| \*\*\*\* |
| --- |

## Slide 6
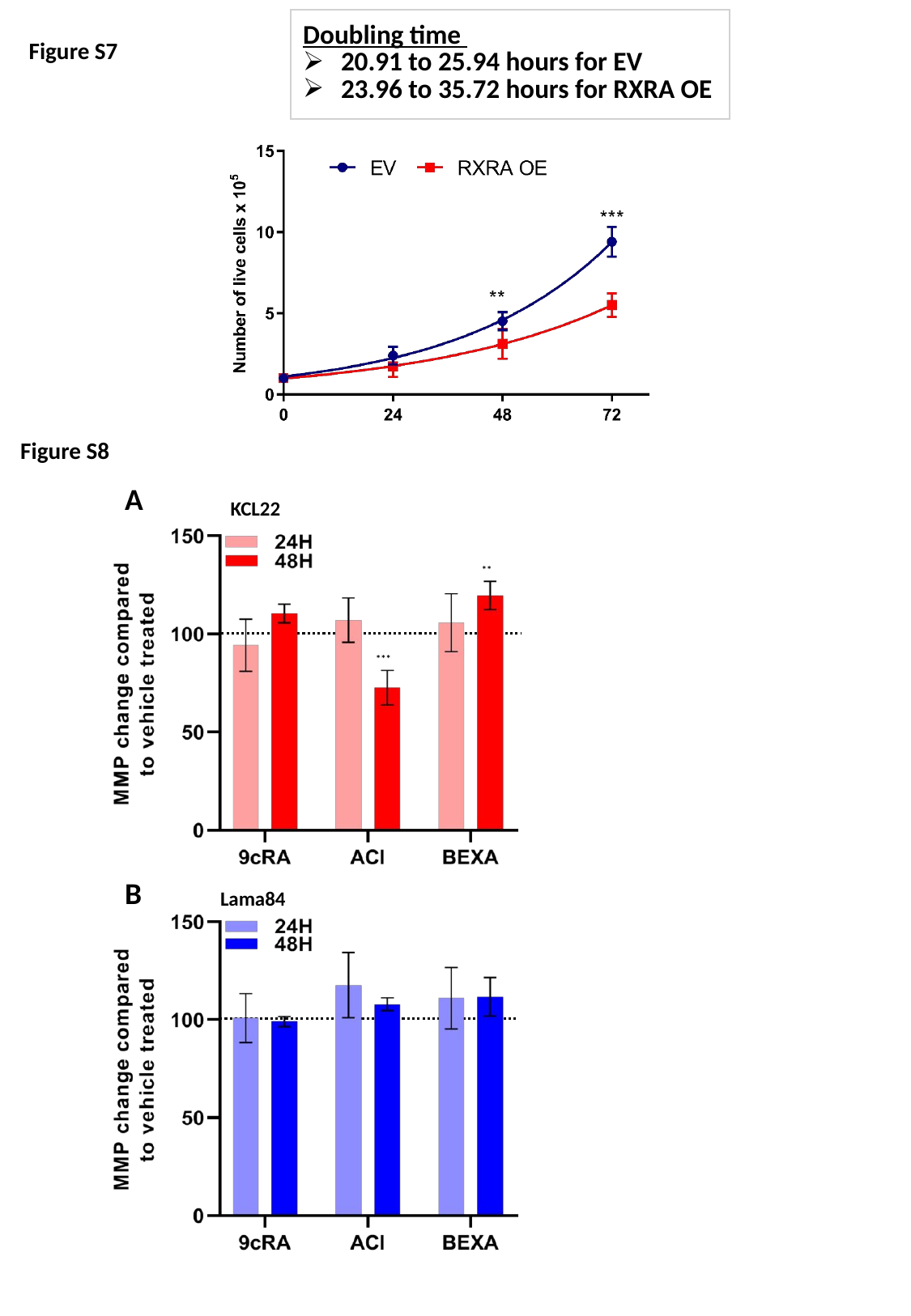

| Doubling time 20.91 to 25.94 hours for EV 23.96 to 35.72 hours for RXRA OE |
| --- |
Figure S7
Figure S8
A
KCL22
**
***
B
Lama84

## Slide 7
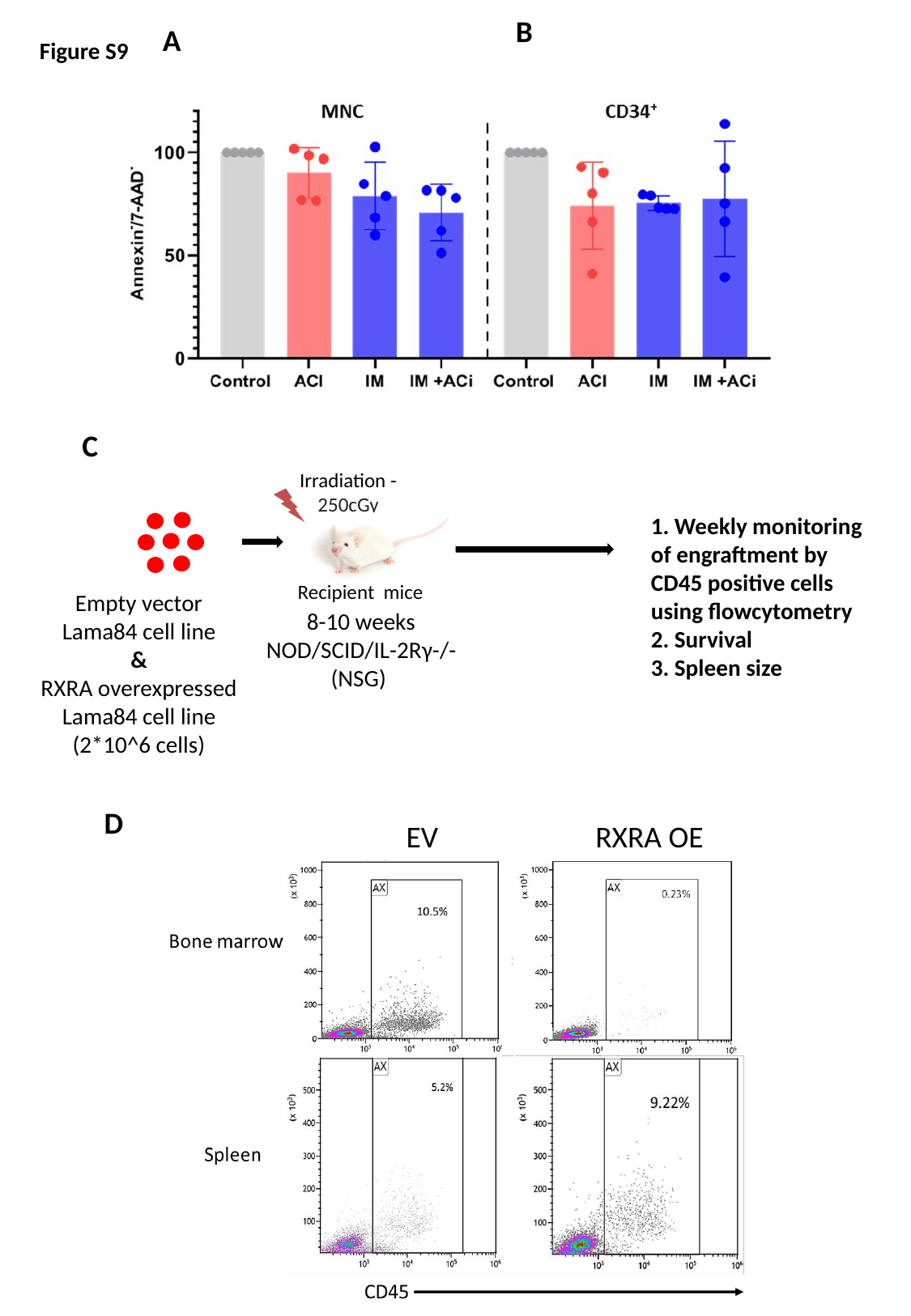

B
A
Figure S9
C
Irradiation -250cGy
Recipient mice
Empty vector Lama84 cell line
&
RXRA overexpressed Lama84 cell line (2*10^6 cells)
8-10 weeks
NOD/SCID/IL-2Rγ-/- (NSG)
1. Weekly monitoring of engraftment by
CD45 positive cells using flowcytometry
2. Survival
3. Spleen size
D
EV
RXRA OE
