# Supplementary material for: Modulating retinoid-X-receptor alpha (RXRA) expression sensitizes chronic myeloid leukemia cells to imatinib in vitro and reduces disease burden in vivo
Source: Front Pharmacol. 2023 May 31;14:1187066. doi: 10.3389/fphar.2023.1187066 (PMC10264673; doi:10.3389/fphar.2023.1187066)
Supplement: Supplementary file 2 [file DataSheet1.pdf]

Supplementary Material

Modulating Retinoid-X-Receptor Alpha (RXRA) expression sensitizes chronic myeloid leukemia cells to imatinib in-vitro and reduces disease burden in-vivo

Bharathi M Rajamani[1], Raveen Stephan Stallon Illangeswaran[1], Esther Sathya Bama Benjamin[1], Balaji Balakrishnan[1*], Daniel Zechariah Paul J[1], Saswati Das[1], Aswin Anand Pai[1], Rakhi TV[1], Ajith Mohan[1], Sreeja Karathedath[1], Aby Abraham[1], Vikram Mathews[1], Shaji R. Velayudhan[1, 2], Poonkuzhali Balasubramanian[1]

*** Correspondence:** Poonkuzhali Balasubramanian, Professor, Department of Haematology,

Christian Medical College, Vellore -bpoonkuzhali@cmcvellore.ac.in

Supplementary table 1: List of NHR ligands and concentrations used.

Supplementary Figure 1: Heatmap representation of basal RNA expression profile of NHRs & coregulators in CML cell lines normalized with five housekeeping genes (*ACTB*, *GAPDH*, *B2M*, *HGPRT* & *RPLP0*). Data were analyzed using the SA biosciences web-based analysis tool, and 2^-dCT was calculated.

Supplementary Figure 2: Primary CML cells pretreated with NHR ligands (A) ATRA, (B) 17-b-estradiol, (C) Pioglitazone, (D) Triiodothyronine, and (E) 9cis retinoic acid for 24 hours followed by imatinib treatment for 48 hours and in-vitro cytotoxicity was assessed, and the change in IC-50 between imatinib alone vs. in combination with ligand was calculated using paired t-test.

Supplementary Figure 3: Primary CML cells were treated with RXRA ligands, and the IC50 to imatinib was compared between those who achieved MMR (n=14) and those who did not achieve MMR (n=16) at 12 months. P-value was calculated by Tukey's multiple comparison test

Supplementary Figure 4: Effect of RXRA ligands in healthy donor cells. PBMNCs (n=4) were treated with (A) 9cRA, (B) Bexa, and (C) ACI for 24 hours, followed by imatinib for 48 hours; in-vitro cytotoxicity assay was performed.

Supplementary Figure 5: Fold change in the RNA expression of *RARA*, *VDR*, and *PPARG* in primary CML cells treated with Aci n=12, 9cRA n=14 & Bexa n=12 compared to untreated cells. The p-value was calculated by Tukey's multiple comparison test.

Supplementary Figure 6: (A) KCL22 and (B) Lama84 cell lines were treated with RXRA ligands for 24 hours, followed by 2nd generation TKIs dasatinib and nilotinib for 48 hours and the percentage apoptosis (Annexin-V and 7AAD positive cells) was measured using flow cytometry. P-value calculated by Tukey's multiple comparison test.

Supplementary Figure 7: The number of viable cells in Lama84 EV vs. RXRA OE cells was assessed using trypan blue exclusion assay (n=3) at three different time points. The doubling time was calculated using exponential analysis, and the p-value was calculated using the Mann-Whitney U test.

Supplementary Figure 8: The quantitative measure of mitochondrial membrane potential using the ratio of JC-1 dimer by JC-1 monomer in RXRA ligand treated CML cell lines (A) KCL22 (B) Lama84. Values represented as percentage change in MMP compared to vehicle treated.

Supplementary Figure 9: Effect of acitretin in healthy donor (A) PBMNCs and (B) $CD34^{+}$ cells (n=5) apoptosis assay was performed. The ppercentage of live cells (Annexin-V and 7AAD negative cells) was measured using flow cytometry. (C) Outline and Development of RXRA overexpression in-vivo xenograft CML mouse model. (D) Representative flow plot for human CD45 expression in the bone marrow and splenic cells from EV and RXRA OE cells
